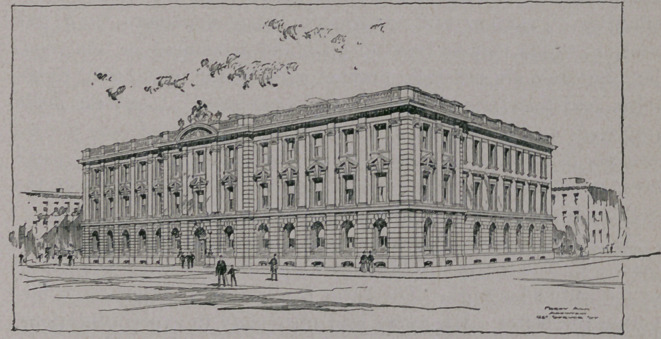# New Medical Laboratories at the University of Pennsylvania

**Published:** 1899-02

**Authors:** 


### New Medical Laboratories at the University of Pennsylvania.

There has been a great deal of agitation in the past about Higher Medical Education, and the theme may seem to have been worn threadbare. With the establishment of the compulsory four years' course of study the ends of the agitators were for a time achieved, and the higher medical education sought after in the past was attained. More years and longer terms of study, with increased practical instruction, completed and rounded out the medical curriculum. To attain this end an additional year of study was urgently necessary, and new clinical facilities had to be established; in short, provision was required to give the student more ample practical experience than had been possible before.

But the world moves swiftly, and another higher medical education has made its appearance. It is true that with the present provisions the University offers a course of study complete in its details, covering the required branches, and covering them far better than ever before, but the foundation of the structure is too narrow, the building is too high for the breadth of base, and the equilibrium is uncertain. The needs of the present time are: more thorough training of the student in the fundamental branches, a greater devotion to laboratory work, and a weeding out of much of the old-fashioned didactic teaching. The hand writing is plainly written on the wall: the laboratory in the future shall be the medium of education, and institutions shall be judged by their laboratory equipment. There was a time, unfortunately not so many years ago, when medical schools in this country graduated doctors of medicine whose whole teaching had been theoretical, on account of the lack of hospital facilities. In those days the stamp by which a medical school could best be measured was the kind and amount of hospital facilities it offered the student. While the importance of hospital training and bed-side instruction is as great as ever, a change of ground has taken place, and laboratory training has become more and more necessary. It is no longer the crucial question whether a certain school offers its students practical instruction, as well as didactic lectures; the decisive test is rather, whether laboratory training is commensurate with the other forms of teaching. It is impossible to teach chemistry in didactic lectures, or even by class demonstrations. This has long been recognized. It is equally impossible to teach anatomy, pathology, histology, or physiology, by didactic lectures, or demonstrations to large classes of students. Individual work is as absolutely essential in these branches at the present time as it has been in the case of chemistry for many years. The scientific physician of today recognizes the all-importance of laboratory study of his case, and will naturally send his students to that institution which offers the most thorough preparation in these branches.

It must not be thought that the University has been entirely remiss. The growing necessity of this form of instruction has been recognized and temporary provision has been made. The physiological laboratory has for several years been conducted upon the principle that each student should receive individual instruction in practical work, using instruments of precision at least sufficiently to attain some command of this sort of work. In histology, in pathology, and in experimental therapeutics, the laboratory facilities have been sufficient up to recent years, while in bacteriology the ample equipment of the department of hygiene has been called in requisition. The continued growth of the classes, however, and the increasing importance of laboratory instruction, make a departure absolutely imperative, and a new laboratory building will have to be provided in the very near future.

The alumni will recognize this need, and will undoubtedly endorse its immediate consideration with unanimous vote. The provost, with characteristic energy, has undertaken the task of securing the means necessary to build and equip new laboratories; and naturally, as in the case of the Law School, turns at once to the alumni of the department. Every alumnus of the Medical School should be reached, and every loyal alumnus will undoubtedly give some amount to this fund. The cost of the building and equipment will be very great; no less than $300,000 will be required for the building and preliminary equipments; and it is urgently hoped that a large part of this fund will be secured by contributions from the alumni. Already the provost has assurances of liberal contributions from the alumni in Philadelphia, and from others, living in remote sections of the country, whose interest in the Alma Mater has not been diminished by their distance from Philadelphia; and wherever an alumnus may be, the mere mention of this project should be received by him as an urgent appeal.

The University of Pennsylvania has ever been the first in suggestion, in preparation, and in execution, looking to the elevation of the tone of medical education in this country; and it is confidently hoped that this latest departure will receive the warmest support from those who in the past have derived so much from the advanced standing of the institution in their student days.

The accompanying plans will illustrate far better than words the character and scope of the proposed undertaking. The building has three floors, devoted to physiology, pharmacology and pathology respectively. The main part of the laboratory building is 192 feet in length, and there are wings running back at each end 128 feet. Large student-laboratories are placed in the wings, while the front of the main building is divided into a number of special research-rooms, offices, and work-rooms of various sorts. To the rear of the center of the main building there is on each floor a special demonstration-room, a lecture-room arranged in the form of an amphitheatre. The animal house is a special building two stories high, and situated at the open side of the hollow square formed by the main building and the two wings.

## Figures and Tables

**Figure f1:**